# Machine learning predictions from unpredictable chaos

**DOI:** 10.1098/rsif.2025.0441

**Published:** 2025-10-01

**Authors:** Jian Jiang, Long Chen, Lu Ke, Bozheng Dou, Yueying Zhu, Yazhou Shi, Huahai Qiu, Ben-gong Zhang, Tianshou Zhou, Guo-Wei Wei

**Affiliations:** ^1^ Research Center of Nonlinear Science, School of Mathematics and Statistics, Wuhan Textile University, Wuhan, Hubei 430200, People’s Republic of China; ^2^ Department of Mathematics, Michigan State University, East Lansing, MI 48824, USA; ^3^ Key Laboratory of Computational Mathematics, Guangdong Province, and School of Mathematics, Sun Yat-Sen University, Guangzhou 510006, People’s Republic of China; ^4^ Department of Electrical and Computer Engineering, Michigan State University, East Lansing, MI 48824, USA; ^5^ Department of Biochemistry and Molecular Biology, Michigan State University, East Lansing, MI 48824, USA

**Keywords:** chaotic systems, machine learning, multiscale topology

## Abstract

Chaos is omnipresent in nature, and its understanding provides enormous social and economic benefits. However, the unpredictability of chaotic systems is a textbook concept due to their sensitivity to initial conditions, aperiodic behaviour, fractal dimensions, nonlinearity and strange attractors. In this work, we introduce, for the first time, chaotic learning, a novel multiscale topological paradigm that enables accurate predictions from chaotic systems. We show that seemingly random and unpredictable chaotic dynamics counterintuitively offer unprecedented quantitative predictions. Specifically, we devise multiscale topological Laplacians to embed real-world data into a family of interactive chaotic dynamical systems, modulate their dynamical behaviours and enable the accurate prediction of the input data. As a proof of concept, we consider 28 datasets from four categories of realistic problems: 10 brain waves, four benchmark protein datasets, 13 single-cell RNA sequencing datasets and an image dataset, as well as two distinct chaotic dynamical systems, namely the Lorenz and Rossler attractors. We demonstrate chaotic learning predictions of the physical properties from chaos. Our new chaotic learning paradigm profoundly changes the textbook perception of chaos and bridges topology, chaos and learning for the first time.

## 1. Introduction

Chaos in dynamical systems refers to the complex behaviour that emerges from deterministic, yet sensitive complex systems such as double pendulum, social network, turbulent fluid flow, weather systems, etc. [1]. Characterized by a sensitive dependence on initial conditions and aperiodic behaviour, chaos challenges the ability to predict long-term behaviour [2]. Nonlinearity is another key feature of chaos, where interactions between system elements lead to unpredictable and often erratic dynamics. The seminal work of Edward Lorenz in the 1960s [3], particularly his study of the weather model, introduced the notion that chaotic systems are fundamentally unpredictable over long timescales. This insight laid the foundation for chaos theory, marking a shift in the way scientists viewed complex systems. Over half a century, the unpredictability of chaotic systems has been a textbook concept in both theoretical and applied dynamics, shaping research and application in various fields, from meteorology to engineering [4].

Chaos theory and chaotic dynamics have profoundly impacted numerous disciplines by providing a framework for understanding complex and nonlinear phenomena [5]. For example, in meteorology, chaos theory revolutionized weather prediction, highlighting the challenge of long-term forecasts due to sensitive dependence on initial conditions [6]. In engineering, chaotic systems have inspired the design of robust control strategies and fault-tolerant systems, especially in nonlinear circuits and mechanical systems [7]. In biology, the study of chaos has shed light on irregular patterns in physiological processes, such as cardiac rhythms and neural activity, enhancing the understanding of health and disease dynamics [8]. Similarly, in economics, chaotic models have been employed to explain irregular market fluctuations, providing insights into complex financial systems [9]. The unpredictability inherent in chaos also plays a pivotal role in encryption [10] and secure communications [11], where it ensures high levels of randomness and security. Across these fields, chaos theory has underscored the importance of recognizing and leveraging unpredictability to uncover hidden structures and drive innovation from complex systems.

Recently, machine learning (ML) has exploited the deterministic nature of chaos theory to predict certain properties of chaotic systems, such as spatial extent and attractor dimension [12], fluctuations in a chaotic cancer model [13], and the evolution of chaotic dynamical systems [14]. Deep learning (DL) methods have also been used to demonstrate chaotic synchronization [15]. However, using chaotic systems for quantitative prediction remains an unthinkable challenge due to their unpredictability.

In this work, we propose chaotic learning to transform unpredictable chaotic systems into accurate predictors. Our approach uses a novel multiscale topological method, known as persistent Laplacian (PL) [16], to embed realistic data in a family of heterogeneous chaotic systems. More specifically, each data point is modelled as a chaotic oscillator, and its interactions with other oscillators are captured with multiscale Laplacians. The resulting data-embedded chaotic systems exhibit rich chaotic patterns that faithfully manifest the traits of the original data, leading to accurate ML predictions. The proposed topology-enabled predictions from chaos (TEPC) use various chaotic dynamics, such as the Lorenz and Rossler systems, for chaotic learning. Additionally, TEPC may employ different persistent topological Laplacians, including persistent spectral graph (PSG) [16] or persistent sheaf Laplacian [17]. Finally, TEPC has been extensively validated by using a wide variety of benchmark data, including organ-scale brain electroencephalogram (EEG) data classification, molecular-scale protein B-factor prediction, cellular-scale single-cell RNA sequence data classification and general image classification problems. TEPC turns chaotic theory on its head, challenging long-held perception of the unpredictability of chaos. It also bridges the gap between traditional chaos and modern learning algorithms.

## 2. Results

### 2.1. Overview of chaotic learning—topology-enabled predictions from chaos

**Figure 1.**
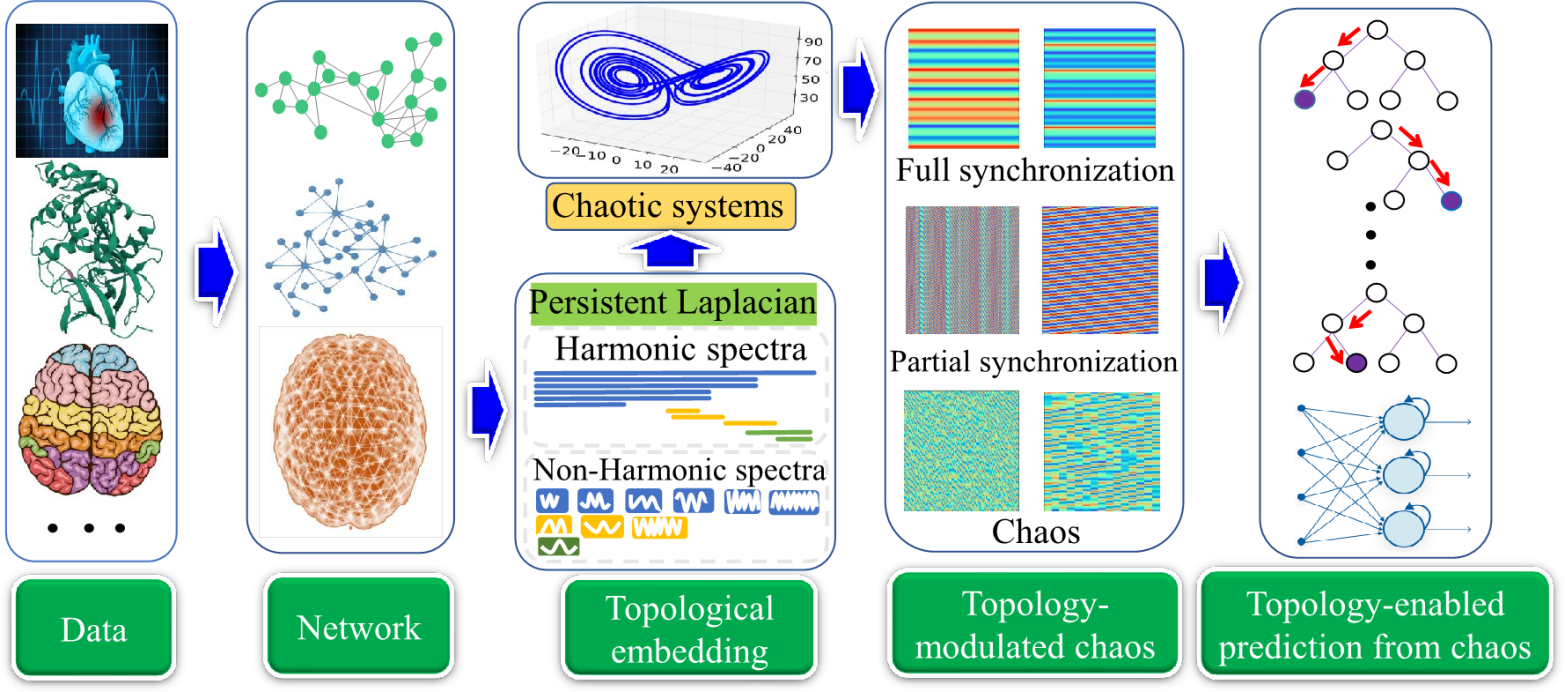
Illustration of the TEPC platform, which provides a general framework to predict the properties of the data of interest, by means of algebraic topology, chaos theory and ML, including DL. First, real-world data is represented by networks where nodes denote individual data points (i.e. atoms, cells, brain nodes, images, etc.) and the links indicate the interaction or the connectivity between nodes. Each node is injected with a chaotic dynamics, like the Lorenz and Rollser oscillator. Then, topological Laplacians, such as PL or persistent sheaf Laplacian, are applied to the networks to encode the multiscale connectivity matrices among nodes, leading to a family of chaotic systems. Subsequently, the evolution trajectories of the topology-regulated chaotic systems are extracted and their statistical features are obtained for various downstream ML/DL tasks. Alternatively, chaotic trajectories are seamlessly fed into a natural language processing (NLP) model for predictions. The brain and heart pictures were adopted from https://www.freepik.com.

Figure 1 illustrates the TEPC platform that provides a general workflow of topology-enabled ML predictions from chaotic dynamics. First, the data of interest are collected from real-world problems, and then an interaction network or a hypergraph is constructed where nodes are individual data points, and the links represent the correlations or connections between nodes. Next, each node is introduced with a chaotic dynamics, such as the Lorenz & Rossler dynamics [18]. A family of chaotic systems is constructed using topological Laplacians [16], which encode the interactions into multiscale connectivity matrices for chaotic systems. Subsequently, the multiscale evolution trajectories of the chaotic systems, which may display various patterns, such as fully synchronized, partially synchronized and unsynchronized chaos, faithfully embed the property of the data of interest. Finally, statistical features are extracted from chaotic trajectories and used for downstream ML or DL predictions. Alternatively, the choice of chaotic trajectories is that these chaotic dynamics is fed into recurrent neural network (RNN) or long short-term memory (LSTM) for downstream predictions as discussed in electronic supplementary material, section S2.

The proposed chaotic learning is an ML algorithm for real-world data prediction rather than a new model for chaos or nonlinear dynamics. Data-embedded chaotic systems have heterogeneous nodal dynamics. Even if complete synchronizations may eventually be achieved, the heterogeneous synchronization process of individual nodes reveals the intrinsic properties of individual nodes, which forms the foundation of the proposed chaotic learning model.

### 2.2. Illustration of chaotic learning principle

**Figure 2.**
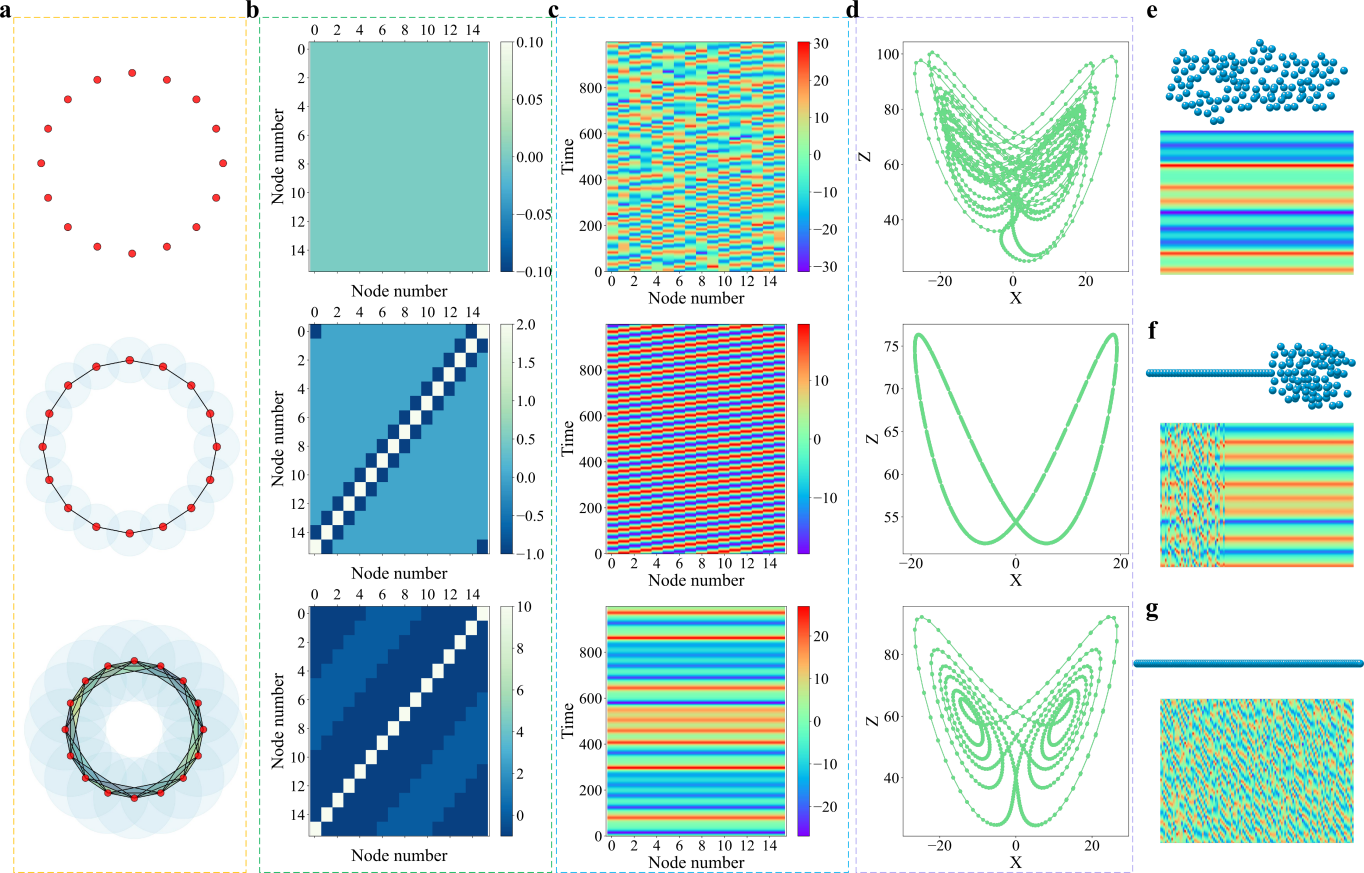
Illustration of PL-induced transition from chaos to periodicity. (a) Each of the 16 nodes in a regular hexadecagon is injected with a Lorenz oscillator. The coupling or connectivity of these nodes are given by the filtration radius of PL. Three typical filtration patterns are displayed in three charts. (b) The connectivity matrices for the coupled chaotic systems are generated by PL at three distinct filtration radii shown in three charts. (c) The trajectories of the coupled chaotic oscillators with random initial values, showing chaos (top), partial synchronization (middle) and full synchronization (bottom). (d) Irregular orbit, periodic orbit and butterfly wing patterns for three chaotic systems. X and Z represent two of the three dynamical variables from the Lorenz oscillator system. The details of Lorenz system equations can be found in electronic supplementary material, equation S3. (e) The folded geometry and its synchronous dynamics of a 120-element point cloud. (f) The partially folded geometry and its partially synchronized dynamics of a 120-element point cloud. (g) The unfolded geometry and its chaotic dynamics of a 120-element point cloud.

To illustrate the working principle of the proposed chaotic learning paradigm, let us consider two simple models. Our first model is a simple model with 16 nodes as shown in figure 2a to demonstrate the proposed paradigm. The three-dimensional coordinates of each node are given in electronic supplementary material, table S13. Each point or node can be regarded as a data point or an individual object, such as an atom in a molecule, a protein in a protein–protein network, or a neuron in a brain network. To extract the localized properties of the 16 nodes, we introduce a chaotic oscillator, or a nonlinear chaotic dynamic to each node. More specifically, we use the Lorenz oscillator although any other chaotic oscillators can be used as well. The PLs are constructed for the set of oscillators, resulting in a family of connectivity matrices for coupled chaotic systems. Three typical connectivity matrices are given in figure 2b, corresponding to three different filtration radii given in figure 2a. The details of these chaotic systems can be found in electronic supplementary material, section S1. The initial values for all oscillators are randomly chosen. The smaller the filtration radius, the narrower the coupling among oscillators is. For example, with the filtration radius shown at the top of figure 2a, since there is no overlap between the nodes, Laplacian is zero everywhere, as shown at the top of figure 2b. Consequently, we have a set of isolated chaotic dynamics as shown on the top of figure 2c, resulting in irregular orbits on the top of figure 2d.

As the filtration radius increases, as shown in the middle of figure 2a, there are some interactions in the system given by the Laplacian in the middle of figure 2b, which causes partial synchronization among chaotic oscillators during the temporal evolution of system (see the middle of figure 2c) and periodic orbit in the middle of figure 2d. When the filtration radius continuously increases, the coupling among the nodes becomes sufficiently strong to create a full synchronization in the system, and each oscillator resembles the well-known wings of butterfly, as plotted at the bottom of figure 2d. In this simple model, it is found that multiscale topological Laplacians induce the transition from chaos to periodicity. However, since the geometry of the above example is very regular, their dynamics is homogeneous, as shown in figure 2a–d.

In our second model, we consider three sets of irregular geometries as shown in figure 2e–g to illustrate how irregular geometries induce heterogeneous chaotic dynamics, including partially synchronized chaos. We create a set of 120 nodes as shown at the top of each chart. The detailed coordinates are given in electronic supplementary material, tables S14–S16, respectively. The geometric shapes of these nodes are schematically displayed in figure 2e–g. We inject the Lorenz dynamics into each of these nodes with the same parameters as those in the early example. However, for chaotic systems, we set the coupling strength as ϵ= 1.1, 1.0 and 0.1 for figure 2e–g, respectively. The connectivity matrices are generated using the PL algorithm with the filtration radii being 1, 1 and 0.2 for the point cloud in figure 2e–g, respectively. We used the forward Euler scheme with the time increment of $h=10^{-3}$ for the time integration. All details of parameters used in figure 2 can be found in electronic supplementary material, table S1.

The first set of 120 nodes is folded, which leads to a fully synchronized chaotic system as shown in figure 2e. This synchronous chaos is very similar to that in the bottom of figure 2c. In figure 2f, the partially folded conformation has 40 unfolded nodes and 80 folded ones, which gives rise to a partially synchronized dynamic behaviour. In figure 2g, the dynamics of the completely unfolded conformation is completely chaotic. It is this strong geometry-dynamics correlation as well as the rich behaviour in chaos synchronization that lays the foundation to embed a wide variety of real-world data into chaotic systems and extract the full set of seemingly chaotic information for the accurate prediction of the underlying data.

Although the filtration radius can be considered a threshold used to define a family of networks, it is not highly sensitive between different applications. This is because we implement a topological filtration procedure on the weighted Laplacian matrix to construct a family of subgraphs or networks, which encode the interactions between oscillators into the multiscale connectivity of the networks. The resulting multiscale evolution trajectories of these networks exhibit various patterns, such as synchronized, partially synchronized and unsynchronized chaos, that effectively capture intrinsic traits of the data for downstream ML predictions. Consequently, in our model, the threshold does not require fine-tuning; an ‘educated choice’ is sufficient to achieve good predictive performance.

### 2.3. Chaotic learning prediction of brain electroencephalogram data

To present a proof-of-principle exploration of the proposed chaotic learning paradigm and demonstrate its predictive power, we consider brain EEG datasets. The EEG is obtained from a medical measurement of brain electrical activity using a number of electrodes applied to the scalp. It plays a significant role in the diagnosis of various patient conditions, such as epilepsy, schizophrenia, sleep disorders and brain tumours. In our analysis, the EEG data have 300 signals and were collected from five epilepsy patients and five healthy individuals, classified into three distinct categories: normal, preictal and seizure [19]. Each category contains 100 single-channel EEG signals. This dataset can be accessed from the official website of the Department of Epileptology of the University of Bonn (https://www.ukbonn.de/epileptologie/arbeitsgruppen/ag-lehnertz-neurophysik/downloads/). Their details can be found in electronic supplementary material, section S5.

**Figure 3.**
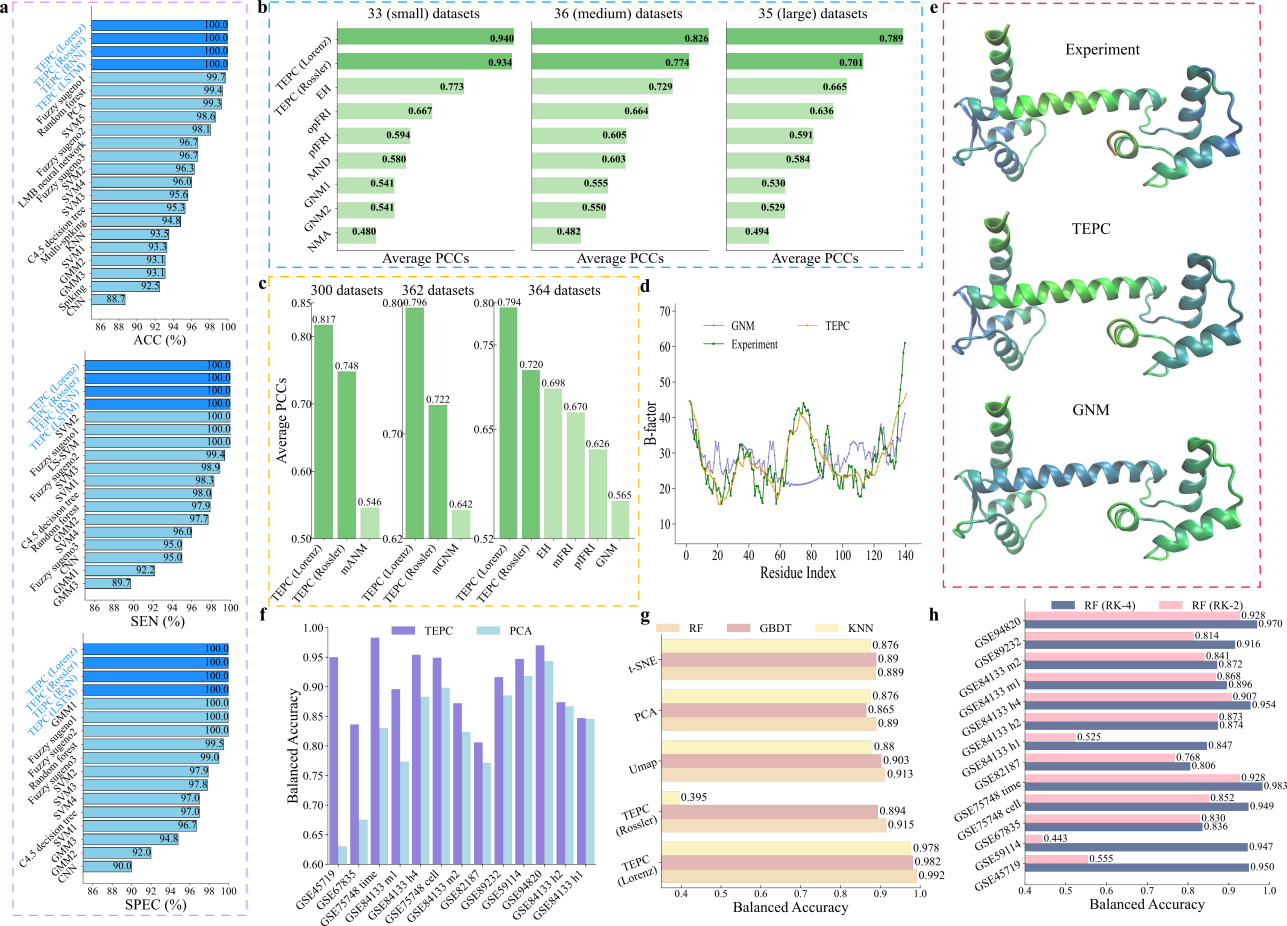
The comparison of prediction results between TEPC method and other existing methods on different types of data, including brain EEG signals, B-factors of proteins, single-cell RNA sequences and image classification. (a) The comparison of brain EEG data classification among TEPC methods (i.e. TEPC (Lorenz), TEPC (Rossler), TEPC (RNN) and TEPC (LSTM)) and other existing methods. The classification involves three categories: normal, preictal and seizure. ACC, SEN and SPEC refer to the accuracy, sensitivity, and specificity, respectively. (b,c) The comparison of B-factor predictions between our TEPC methods with Lorenz and Rossler oscillators and other literature methods on three datasets, on a benchmark dataset of 300, 362 and 364 proteins. (d) The comparison of TEPC, Gaussian network model (GNM) prediction value and experimental value for each residue. The x-axis represents the residue index, and the y-axis represents the B-factor. (e) The three-dimensional structures of protein calmodulin (PDB ID: 1CLL), coloured by experimental B-factors, TEPC predicted B-factors and GNM method predicted B-factors from top to bottom, respectively. (f) The comparison of the balanced accuracy between our TEPC method with Lorenz oscillators and principal components analysis (PCA) method through an RF classifier on 13 single-cell RNA sequence data. (g) The comparison of balanced accuracy between our TEPC method with Lorenz and Rossler oscillators and UMAP, PCA, t-SNE method with RF, gradient boosting decision tree (GBDT) and k-nearest neighbour classifier (KNN) on Coil-20 image data. (h) The comparison of balanced accuracy between fourth-order Runge–Kutta (RK−4) and second-order Runge–Kutta (RK−2) by our TEPC method with Lorenz oscillators through a RF classifier.

We embed the real brain activity time series data into nonlinear dynamics systems by considering each EEG signal as a nonlinear oscillator. We further compute the mean coupling matrix of this brain neural network by calculating the Pearson correlation coefficient between each pair of 300 signals, respectively. We then apply PL to obtain a family of connectivity matrices of brain neural networks with the filtration process. For each connectivity matrix, we introduce a chaotic system and collect its trajectories. Their statistical values are computed for EEG classification. To demonstrate the predictive power of TEPC, we consider classification predictions of the EEG data, where 10-fold cross-validation with 10 random seeds and k-nearest neighbour classifier (KNN) algorithm were used in the prediction. The details of parameters used in the implementation are given in electronic supplementary material, table S2. Our TEPC was implemented with two different oscillators, i.e. Lorenz and Rossler, and two neural network models, i.e. RNN and LSTM, denoted as TEPC (Lorenz), TEPC (Rossler), TEPC (RNN) and TEPC (LSTM), respectively. Their comparison with other existing methods is shown in figure 3a, including fuzzy sugeno1 [20], random forest (RF) [21], principal components analysis (PCA) [22], SVM5 [23], fuzzy sugeno2 [24], LMB neural network [25], fuzzy sugeno3 [26], SVM2 [27], SVM4 [28], SVM3 [29], C4.5 decision tree [30], Multi-spiking [31], KNN [32], SVM1 [33], GMM2 [34], GMM3 [35], Spiking [36] and CNN [37]. ACC, SEN and SPEC in figure 3a are accuracy, sensitivity and specificity, respectively. From figure 3a, we can find that TEPC (Lorenz), TEPC (Rossler), TEPC (RNN) and TEPC (LSTM) obtain all 100% on these three metrics, which are equivalent to or higher than those of other existing methods. The confusion matrices for all 10 folds of these results are given in electronic supplementary material, tables S8–S11. These results indicate that TEPC is a general approach that is robust with respect to the selection of dynamic models and ML algorithms.

### 2.4. Predicting protein B-factors from chaotic systems

As another proof of principle, we demonstrate quantitative ML predictions of molecular properties of chaotic systems. To this end, we consider protein B-factor predictions. The protein B-factor or the Debye–Waller factor is used in protein crystallography to describe the attenuation of X-ray caused by thermal motion. The analysis of protein B-factors sheds light on the large-scale and long-time functional behaviours of native state macromolecules [38,39]. There are many existing methods for protein flexibility analysis. For example, Park *et al.* [39] compared the performance of normal mode analysis (NMA) [40] and Gaussian network model (GNM) [38] for three sets of proteins. Among them, the GNM is the most popular method for protein flexibility analysis [38]. Xia *et al.* developed generalized GNM and generalized anisotropic network model (ANM) for protein B-factor predictions [41]. Opron *et al.* proposed a multiscale flexibility rigidity index (FRI) model to achieve nearly 20% improvement to the GNM prediction [42]. Recently, Cang *et al.* introduced an evolutionary homology method to analyse the protein flexibility and obtain state-of-the-art performance [43].

In the present work, we use a few benchmark datasets that have been intensively studied in the literature [39,43,44], and thus, the performance of the proposed methods can be compared. The largest dataset involves 364 distinct proteins, having tens to thousands of amino acid residues [44].

In the proposed approach, each protein residue is represented by its C${,}_{\alpha }$ atom. Each C${,}_{\alpha }$ atom is assigned with a nonlinear oscillator. We choose the Lorenz system with parameters α=10, γ=60, β=8/3 and ϵ=13.6 with the connectivity matrix being Γ=I (the identity matrix). The interaction strength between each pair of C${,}_{\alpha }$ atoms is computed with the generalized exponential function (equation (4.2)) with κ=1 and σ=3. We further use PLs to obtain a family of coupling matrices among the residues at various scales according to filtration. At each scale, we have a coupled chaotic system. To generate the time trajectory of a specific oscillator (residue), we choose its initial value as one and those of other oscillators as zero in a perturbative time integration. Then, the statistic values of the trajectory are used as the inputs in the ML study of the residue’s B-factor. PLs enable us to collect multiple sets of the statistic values for each residue.

We carry out B-factor predictions by a multiple linear regression model that has been used in earlier papers [38,39,44] and compute the average Pearson correlation coefficients (PCCs) between the predicted and the experimental B-factors. Figure 3b,c shows a comparison of average PCCs over different datasets with other existing methods, including EH [43], opFRI [44], pfFRI [44], MND [45], GNM [39,45], NMA [39], mFRI [42], mGNM [41] and mANM [41]. Here, TEPC (Lorenz) and TEPC (Rossler) are the present methods implemented through the Lorenz oscillator and Rossler oscillator, respectively. We find that our method TEPC (Lorenz) outperforms all other methods in computational biophysics and achieves the largest average PCCs for all different protein datasets. Specifically, in figure 3b, for three sets of proteins with small (33 samples), medium (36 samples) and large (35 samples) sizes, our average PCCs are 0.940, 0.826 and 0.789 by TEPC (Lorenz), which are improved up to 95.8%, 71.4% and 59.7% compared with those of 0.480, 0.482 and 0.494 in [39], respectively. Furthermore, in figure 3c, compared with the best existing results, the average PCCs 0.794, 0.796 and 0.817 obtained by TEPC (Lorenz) are approximately 13.8%, 24.0% and 49.6% higher than 0.698 [43], 0.642 [41] and 0.546 [41] in 364, 362 and 300 datasets, respectively. The potential reason for this excellent performance is that the proposed method provides richer information of residue interactions through multiscale topological Laplacians. More details about the results in figure 3b,c and the prediction result of B-factor on 364 datasets can be found in electronic supplementary material, tables S12 and S17, respectively. Three examples of the B-factor predictions of proteins 1FF4, 2WUL and 2Y7L with correlation coefficients 0.963, 0.955 and 0.891 are given in electronic supplementary material, figure S6, which suggests that our prediction results agree well with experiments.

Figure 3d,e shows another B-factor prediction on protein calmodulin (PDB ID: 1CLL). In figure 3d, our TEPC method with the Lorenz oscillator displays excellent performance, and its results match closely with experiments for residues 60−90. In contrast, the GNM method demonstrates poor predictions. This difference is obvious when comparing the colour representations based on B-factor values of the 1CLL protein structure in figure 3e.

These protein B-factor predictions not only demonstrate the usefulness of the present TEPC method in handling proteins with different sizes and different complex structures, but also indicate that the proposed approach has great potential for other important biophysics applications, such as the predictions of protein–ligand binding affinities and protein–protein interactions.

### 2.5. Predicting single cell properties from chaotic systems

Having demonstrated the utility of topology-enabled molecular property predictions from chaos, we further demonstrate the topology-enabled cellular property predictions from chaos. Specifically, we consider single-cell RNA sequencing (scRNA-seq) data analysis, which is a computational process aimed at extracting meaningful biological insights from single-cell gene expression data. In the present work, we employ 13 scRNA-seq datasets as shown in electronic supplementary material, table S4. These datasets involve tens of thousands of single cell samples collected from humans or mice. The single cells contain thousands or tens of thousands of genes. The problem is to classify these cells into various cell types, from 4 to 14 types, in various datasets based on their gene expressions.

We first compute the Euclidean distance between cells and then set κ=1 and σ=3 for the exponential kernel in equation (4.2) to evaluate pairwise correlations. We employ PLs to create a family of correlation matrices for various chaotic systems. The trajectories from chaotic systems are collected, and their statistical values are computed for cell classification. We use the fivefold cross-validation with 10 random seeds and the RF algorithm to predict cell type. The default parameters of RF from Scikit-learn’s packages were used in the implementation. Here, we compare the overall classification performance of our method (TEPC with Lorenz oscillators) with the widely used PCA method in figure 3f.

For PCA, we take the average value of balanced accuracy (BA) over different dimensions, namely 50, 100, 150, 200, 250 and 300. For each reduction, 20 random initializations were used to ensure robustness and stability. Additionally, before the dimensionality reduction, a log-transform was performed on the datasets. The five-fold cross-validation is employed to assess the performance of various methods. Since scRNA-seq datasets usually have an unbalanced number of cells per type, we use a balanced accuracy score rather than the traditional accuracy score, whose details are given in electronic supplementary material, section S4. From figure 3, we find that our TEPC with Lorenz oscillators outperforms PCA on all 13 scRNA-seq datasets. Specifically, the highest improvement of BA value is up to 50.79% on GSE45719 data and the average improvement is 11.29% for each dataset, suggesting that TEPC is a useful tool in the classification of scRNA-seq data. The details of figure 3f can be found in electronic supplementary material, table S5.

### 2.6. Predicting image properties from chaotic learning

To further demonstrate the predictive power and robustness of the proposed TEPC, we consider a non-biological problem. Image classification is a general task in data science and has widespread applications in a wide variety of fields and disciplines. In this work, we consider Coil-20, a benchmark dataset of image classifications with 1440 images [46]. Each image has a size of 128 × 128, where 20 objects or classes are captured at 72 angles, and was treated as a vector of length 16 384. The details of the Coil-20 data are given in electronic supplementary material, section S7. In our implementation, we first reduce the dimension from 16 384 to 3 with uniform manifold approximation and projection (UMAP). However, t-distributed stochastic neighbour embedding (t-SNE) or PCA could be similarly used for dimension reduction. The pairwise correlations among the images in the dataset are computed with the exponential kernel κ=1 and σ=3 from the three-dimensional UMAP results. Then, we use PLs to generate a family of correlation matrices for 1440 images. For each correlation matrix, we install a chaotic system and compute a perturbative trajectory for each image. The statistical values of the trajectories are used for downstream ML predictions. For comparison, we also directly use the features from the UMAP, PCA and t-SNE dimension reduction for downstream classifiers. We use three different classifiers, including RF, gradient boosting decision tree (GBDT) and KNN in the classification with all default parameters of Scikit-learn packages. We use the fivefold cross-validation to evaluate the performance of classifications. All details of the parameters used in figure 3 can be found in electronic supplementary material, table S2.

In figure 3g, we compare the balanced accuracy of classification with these four methods, i.e. TEPC, UMAP, PCA and t-SNE, where TEPC (Lorenz) and TEPC (Rossler) indicate the use of two different chaotic dynamics, i.e. Lorenz and Rossler oscillators. We find that TEPC (Lorenz) outperforms UMAP, PCA and t-SNE methods on all three classifiers. Compared with UMAP, PCA and t-SNE, TEPC (Lorenz) has improved the balanced accuracy up to 11.59%, 13.53% and 11.64% for RF, GBDT and KNN classifiers, respectively. The details of figure 3g can be found in electronic supplementary material, table S7. It is interesting to note that although TEPC (Lorenz) started with the same UMAP information, it outperforms UMAP by 8.65%, 8.75% and 11.14% for RF, GBDT and KNN classifiers, respectively. This amazing improvement is due to the fact that the proposed topology-enabled chaotic embedding captures the network interactions and multiscale correlations among oscillators (images). In contrast, the UMAP information is relatively isolated for each image and neglects crucial ‘spatial’ correlations among images.

## 3. Discussion

### 3.1. Robustness with respect to dynamical systems and machine learning models

In order to demonstrate the robustness of the proposed chaotic learning with respect to the choice of chaotic dynamics and ML algorithms, we consider two oscillators, i.e. Lorenz and Rossler and many ML algorithms, including KNN, RF, GBDT, RNN and LSTM. Specifically, we integrate the Rossler equation using the fourth-order Runge–Kutta scheme and set their parameters to a=b=0.1, c=4 and a=35,b=3,c=28*,* respectively. In figure 3, TEPC (Rossler) means the TEPC method with Rossler oscillators.

In figure 3a, TEPC (Rossler) performances are as good as TEPC (Lorenz) on the EEG classification task, obtaining 100% in the three metrics, accuracy, sensitivity and specificity, which are equivalent or higher than those of other existing methods.

In figure 3b, EH indicates the evolutionary homology method, and NMA refers to the normal mode analysis. Regarding the protein B-factor prediction, for the set of small-size proteins with 33 samples, TEPC (Rossler) method with an average PCC 0.934 outperforms other existing methods. Specifically, the average PCC value is improved 20.81% and 94.58% compared with 0.773 and 0.480 obtained by EH and NMA methods in [43] and [39], respectively. For the set of medium-size proteins with 36 samples, TEPC (Rossler) raises the average PCC value up to 6.17% and 60.58% from 0.729, 0.482 to 0.774 compared with those of EH method [43] and [39], respectively. Similarly, for the set of large-size proteins with 35 samples, TEPC (Rossler) raises the average PCC value up to 5.4%, 41.9% compared with those of EH method [43] and reference [39], respectively. Additionally, in figure 3c, TEPC (Rossler) also achieves better prediction results than other existing methods in different sets of proteins with 300, 362 and 364 samples, respectively. That is, TEPC (Rossler) gets the average PCCs 0.748, 0.722 and 0.72 with the increment up to 37.0%, 12.46% and 27.43% compared with those of 0.546, 0.642 and 0.565 by mANM [41], mGNM [41] and GNM methods [44] with 300, 362 and 364 datasets, respectively.

In figure 3g, for image classification task, TEPC (Rossler) obtains a comparable performance with TEPC (Lorenz) method, except for using KNN classifier. The balanced accuracy values, 0.915 and 0.894, for the RF and GBDT classifiers attained by TEPC (Rossler) are improved up to 2.8% and 3.35% compared with the PCA method, and 2.92% and 0.45% for t-SNE method, respectively, which is similar to those of the UMAP method. However, TEPC (Rossler) with KNN classifier performs worse than other methods.

From figure 3a–c,g, although the TEPC method with different kinds of oscillators has various performances on brain EEG data, protein B-factor prediction and image classification task, it outperforms the existing advanced methods or the traditional baseline methods, like PCA or UMAP or t-SNE in most cases, which verifies the generalizability of TEPC method is independent of specific chaotic systems.

Finally, in figure 3a, TEPC (RNN) and TEPC (LSTM) were paired with Lorenz, and TEPC (Lorenz) and TEPC (Rossler) were combined with KNN. All of these methods achieved 100% in accuracy, sensitivity and specificity for EEG classification, confirming that the proposed TEPC method is highly robust.

### 3.2. Impact of numerical integrators

In the implementation of TEPC method, we all use the fourth-order Runge–Kutta (RK-4) method to solve Lorenz (Rossler) equations. Here, in order to discuss the effect caused by different numerical integrators on ML predictions, we show the comparison results between the RK-4 and RK-2 methods on 13 single-cell RNA sequencing data in figure 3h, where the BA is compared by the TEPC method with the Lorenz oscillator integrating with three different classifiers, RF, GBDT and SVM.

From figure 3h, the BA values by RK-4 method are larger than those by RK-2 with RF classifier in 13 single-cell datasets. For the GBDT and SVM classifier, the comparison results between the RK-4 method and the RK-2 method can be found in electronic supplementary material, figure S5. These results suggest that the RK-4 method used in our numerical experiments is a robust approach to provide reliable and efficient prediction results. The details of figure 3h can be found in electronic supplementary material, table S6.

### 3.3. Impact of model parametrization

To analyse the best parameter for the exponential function of equation (4.2), we test a range of parameters to predict the B-factor averaged on protein 6RXN in electronic supplementary material, figure S1, where the best average B-factor is approximately κ=1 and σ=3. This result suggests that the TEPC model introduced in the present study can be a parameter-free model by setting κ=1 and σ=3. Our findings do not depend on chaotic models and model parametrization.

### 3.4. Residue–similarity plots

In this section, we implement the residue–similarity (R-S) analysis [47] for the visualization of classification performance on single-cell RNA sequencing (scRNA-seq) datasets. In classification tasks involving two classes, conventional approaches such as receiver operating characteristic (ROC) curves and area under the ROC curve (AUC) serve comparable purposes to R-S scores. However, unlike these traditional methods, R-S scores possess the capability to be extended to scenarios with any number of classes, which is advantageous over traditional methods.

**Figure 4.**
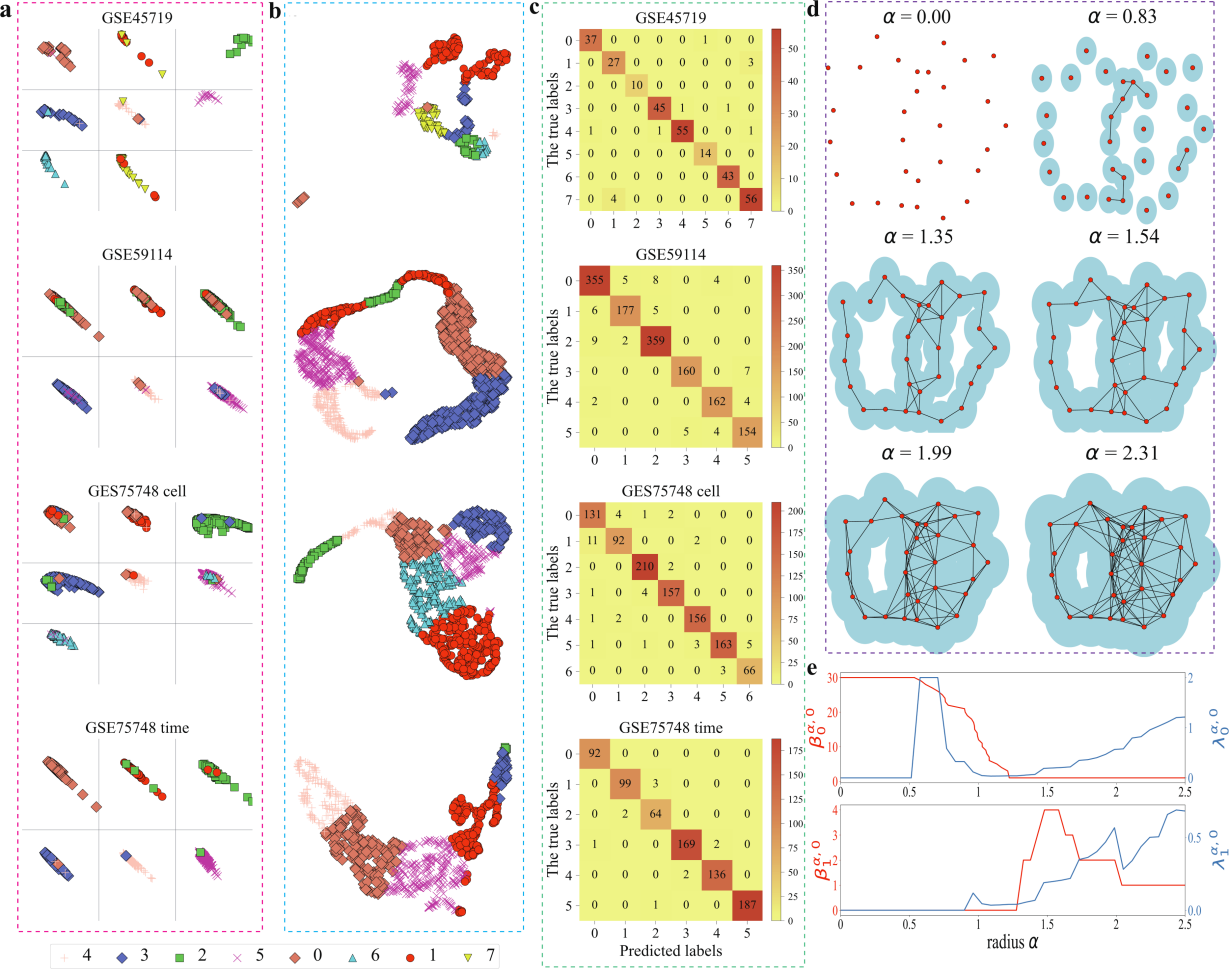
R-S plots, UMAP plots and the confusion matrix of R-S plots of four scRNA-seq datasets, including GSE45719, GSE59114, GSE75748 cell and GSE75748 time datasets. (a) The R-S plots. The TEPC method was used to reduce the scRNA-seq datasets to 30 components with κ=1 and σ=3. Fivefold cross-validation was used to split the data into five parts, where four parts were used for training and one part was used for testing the RF classifier. The R-S score was computed for the testing set, and all five folds were visualized. Each section corresponds to one of the eight true cell types, and the sample’s colour and marker correspond to the predicted label from the RF classifier. (b) The UMAP plots. The data was log-transformed and any genes with less than $10^{-6}$ variance were removed before applying the reduction. Samples were coloured according to their cell types. (c) The confusion matrix based on the classification results of R-S plots. (d) The illustration of filtration process of point clouds with the generation of a series of simplicial complexes. (e) The changes of persistent Betti numbers $\beta_{0}^{\alpha ,0}$, $\beta_{1}^{\alpha ,0}$ and the smallest non-zero eigenvalues of PL $\lambda_{0}^{\alpha ,0}$, $\lambda_{1}^{\alpha ,0}$ with the increasing of filtration radius α, respectively.

Figure 4a,b shows a comparison between the R-S plot and two-dimensional plots of UMAP for four scRNA-seq datasets, GSE45719, GSE59114, GSE75748 cell and GSE75748 time. For each dataset, it was partitioned into five subsets using fivefold cross-validation. Four subsets were used to train the RF classifier, while one subset was reserved for testing. Subsequently, residue and similarity scores were calculated for each sample and visualized based on the basis of their true cell type. Samples were then colour-coded according to the labels predicted by the RF classifier. The x-axis represents residue scores, while the y-axis represents similarity scores. Both scores range from 0 to 1, with 1 indicating optimal performance. A well-separated and clustered reduction is indicated by the top-right corner of the plot. The TEPC model was used to reduce the dimension to 30 supergenes with κ=1 and σ=3.

Figure 4c represents the confusion matrix of the R-S plots, where the x-axis represents the true labels and the y-axis represents the predicted labels. For instance, for the GSE45719 dataset, in the first row of the figure, we can find that classes 2, 5 and 6 are all classified correctly, and classes 0, 1, 3, 4 and 7 are classified correctly except for one, three, three and four samples, respectively. However, in UMAP plots, the samples of classes 0, 1, 3 and 7 do not form compact individual clusters, and there are two obvious separated clusters for each class. These findings suggest that although UMAP has a good clustering visualization, an R-S plot has better prediction performance than UMAP. Similar results are found in the cases of GSE59114, GSE75748 cell and GSE75748 time datasets, shown in the last three rows of figure 4a–c. The clustering visualization of remaining scRNA-seq datasets is given in electronic supplementary material, figures S2–S4.

### 3.5. Applications of chaotic learning to other fields

In the present work, chaotic learning has been applied to diverse complex systems, from single molecules (proteins), single cells, organs (brain EEG), to non-biological data (images). Although all examples shown in this work are about element-specific predictions, the proposed TEPC can be used for global property predictions by appropriately integrating element-specific features into global ones, such as features for a whole protein, which can be used for global predictions, such as protein solubility, toxicity, folding free energy, etc.

Here, by integrating ML, chaos theory and PL, we propose a chaotic learning paradigm to predict the properties of general data from unpredictable chaotic dynamics. Our paradigm can be used not only as a tool in ML predictions, but also as a new method for the analysis of times series, such as the brain EEG data.

Several limitations of the present study should be acknowledged. First, the computational efficiency of the proposed method was not prioritized, given that the primary emphasis was placed on the novelty of the proposed ideas and conceptual framework. Furthermore, the incomplete mechanistic comprehension regarding how the chaos specifically contributes relative to topological embedding in isolation warrants further investigation in future work. Additionally, the proposed chaotic learning can be improved by other topological Laplacian methods, such as persistent hyperdigraph Laplacian and persistent sheaf Laplacian [17], as well as better time integrators.

## 4. Methods

We consider a spatial network or high-order hypergraph where the nodes represent objects, particles or neurons, and the links or edges between nodes represent the interaction between nodes. The distance between two nodes is directly associated with their interaction strength. Additionally, we assume that all functions of nodes are solely determined by the network structure. Therefore, functional predictions can be carried out with the structural information without resorting to the ultimate interactions. We build up a connectivity matrix from the interactive network, and we represent the dynamics of each node by a nonlinear oscillator, and couple nodes with the connectivity matrix extracted from the data. Finally, we review PL and propose a new filtration method, Laplacian filtration, instead of a conventional filtration method. In this section, we discuss the details of our main methods, mapping interaction to topology and PL analysis. A brief discussion of the stability analysis of coupled chaotic systems and the details of R-S scores are given in electronic supplementary material, sections S1.2 and S9, respectively.

### 4.1. Mapping interaction to topology

Topological relations or connectivities among network nodes are basic ingredients in network nonlinear dynamics models. As discussed above, this topological information can be extracted from the interaction of a given node of interest in an n-dimensional space. Let us consider a network of N nodes located on different regions $r_{1},r_{2},\cdots ,r_{N}$*,* ($r_{j}\in R^{n}$), where nodes can be neurons, atoms, amino acid residues in a molecule, or people in society if applied in other scenarios. The distance between the ith and jth nodes is given by $d_{ij}(r_{i},r_{j})={\left\|r_{i}-r_{j}\right\|}_{2}$. The connectivity matrix must agree with the driven and response relation between two dynamics systems. Moreover, we assume that all nodes in the network are mutually linked and their interactions decay as a function of their distance $A_{ij}(d_{ij})$. The simplest form of the connectivity matrix is the Kirchoff (or connectivity) matrix generated by cut-off distance $\sigma_{ij}$,


(4.1) $$A_{ij}=\left\{ \begin{array}{l} 1,\;\forall d_{ij}\leq \sigma_{ij},i\neq j\\ 0,\;\forall d_{ij}>\sigma_{ij},i\neq j\\ -\sum_{j\neq i}A_{ij},\;\forall i=j \end{array}\right..$$

In order to take the distance effect into a more practical way in the consideration, smooth and monotonically decreasing radial basis functions or the delta sequence kernels of positive type are often employed for convenience [48]. Here, generalized exponential functions


(4.2) $$A_{ij}=\left\{ \begin{array}{c} e^{-d_{ij}^{k}/k\sigma_{ij}^{k}},\;\forall i\neq j,k=1,2,\cdots \\ -\sum_{j\neq i}A_{ij},\;\forall i=j \end{array}\right.$$

and power law functions are considered


(4.3) $$A_{ij}=\left\{ \begin{array}{c} {\left(d_{ij}/\sigma_{ij}\right)}^{-v},\;\forall i\neq j,v>1\\ -\sum_{j\neq i}A_{ij},\;\forall i=j, \end{array}\right.$$

where $\sigma_{ij}$ are characteristic distance between nodes. In the present work, $\sigma_{ij}$ is a tuneable parameter. Note that the above matrix functions have already been applied to other methods, like flexibility analysis methods in a biology system [49,50]. However, the present construction of these functional forms is based on the driven response relationship of coupled dynamical systems [51].

Formulae (4.1)–(4.3) can be used to map the geometry of a network into topological relations or connectivities. The connectivity matrix A is an N×N symmetric, diagonally dominant matrix and the elements in the matrix are not interaction potentials among nodes. For simplicity, we set the characteristic distances of all nodes to be the same $\sigma_{ij}=\sigma$.

### 4.2. Persistent Laplacian analysis

Persistent homology (PH) is a new branch of algebraic topology and has been developed as a new multiscale representation of topological features applied successfully in various fields, including mathematics [52], chemistry [53], etc. It contains a filtration process to generate a family of topological spaces so that the system can be analysed at multiscales. More discussion of PH theory is given in electronic supplementary material, section S8. However, PH cannot detect the homotopic shape evolution of the system during filtration. Recently, PL or PSG is developed to fill the gap. PL not only retains the full set of topological invariants in their harmonic spectra as PH does but also captures the homotopic shape evolution of data during the filtration in their non-harmonic spectra that PH does not describe [16]. PL is a special case of persistent topological Laplacians, including the persistent Hodge Laplacian defined on a Riemannian manifold [54], and a variety of other formulations defined on many topological spaces, such as cellular sheaf, hyperdigraphs, path complexes, etc. Like PH, PLs use a filtration process to generate a series of geometric shapes and associated topological spaces, on which PSGs are defined. The change in the null space dimensions of the PLs during the filtration indicates the persistence of topological invariants, while the non-zero eigenvalues and related eigenfunctions of the PLs reveal the geometric shape evolution of the data during the filtration.

An oriented simplicial complex K is a sequence of subcomplexes $(K_{t}{)}_{t=0}^{m}$ of K,


(4.4) $$\begin{array}{lcr} & \emptyset =K_{0}\subseteq K_{1}\subseteq K_{2}\subseteq \cdots \subseteq K_{m}=K. & \end{array}$$

Considering the corresponding chain group of each subcomplex $K_{t}$ and q-boundary operator: $C_{q}(K_{t})$ and $\partial_{q}^{t}:C_{q}(K_{t})\to C_{q-1}(K_{t})$, if $0<q\leq \dim\;K_{t}$, we have


(4.5) $$\begin{array}{lcr} & \partial_{q}^{t}(\sigma_{q})=\sum_{i}^{q}(-1{)}^{i}\sigma_{q-1}^{i},\;\forall \sigma_{q}\in K_{t}, & \end{array}$$

where $\sigma_{q}=\left[v_{0},\cdots \;,v_{q}\right]$ is any q-simplex and $\sigma_{q-1}^{i}=\left[v_{0},\cdots \;,\hat{v_{i}},\cdots \;,v_{q}\right]$ the oriented (q−1)-simplex constructed by removing $v_{i}$. It is important to define an adjoint operator of $\partial_{q}^{t}$ as the co-boundary operator $\partial_{q}^{t^{*}}:C^{q-1}(K_{t})\to C^{q}(K_{t})$, mapping from $C_{q-1}(K_{t})$ to $C_{q}(K_{t})$ by the isomorphism between cochain groups and chain groups $C^{q}(K_{t})≅C_{q}(K_{t})$.

For simplicity, denoting $C_{q}^{t}$ the chain group $C_{q}(K_{t})$ and using the natural inclusion map from $C_{q-1}^{t}$ to $C_{q-1}^{t+p}$, one defines the subset of $C_{q}^{t+p}$ as ${\mathbb{C}}_{q}^{t,p}$ with the boundary in $C_{q-1}^{t}$ as


(4.6) $$C_{q}^{t,p}:=\{\beta \in C_{q}^{t+p}|\;\partial_{q}^{t+p}(\beta )\in C_{q-1}^{t}\}.$$

In this subset, we denote the p-persistent q-boundary operator by ${ð}_{q}^{t,p}:{\mathbb{C}}_{q}^{t,p}\to C_{q-1}^{t}$ and the corresponding adjoint operator by $({ð}_{q}^{t,p}{)}^{*}:C_{q-1}^{t}\to {\mathbb{C}}_{q}^{t,p}$ via the determination of co-chains with chains. We can define the q-order p-persistent Laplacian operator $\Delta_{q}^{t,p}:C_{q}^{t}\to C_{q}^{t}$ in the filtration [16],


(4.7) $$\begin{array}{lcr} & \Delta_{q}^{t,p}={ð}_{q+1}^{t,p}{\left({ð}_{q+1}^{t,p}\right)}^{*}+\partial_{q}^{t^{*}}\partial_{q}^{t}. & \end{array}$$

The matrix representation of $\Delta_{q}^{t,p}$ on the simplicial basis is


(4.8) $$\begin{array}{lcr} & L_{q}^{t,p}=B_{q+1}^{t,p}(B_{q+1}^{t,p}{)}^{T}+(B_{q}^{t}{)}^{T}B_{q}^{t}. & \end{array}$$

The topological invariants of the corresponding PH defined by the same filtration can be recovered from the kernel of PL (equation (4.7)),


(4.9) $$\begin{array}{r} \beta_{q}^{t,p}=\dim\ker\partial_{q}^{t}-\dim\text{im}{ð}_{q+1}^{t,p}=\dim\ker L_{q}^{t,p}=\#\text{0 eigenvalues of }L_{q}^{t,p}. \end{array}$$

In this study, we focus on the zero-, first- and second-order PLs for the brain neural network data. Mathematically, $\beta_{q}^{t,p}$ from the null space of LPs tracks the number of independent q-dimensional holes in $K_{t}$ that are still alive in $K_{t+p}$. Therefore, it gives the same topological information as PH does. However, the non-zero eigenvalues of the PLs reveal the homotopic shape evolution of the data during filtration.

It is worth noting that rather than conventional filtration method directly by studying the simplicial complexes themselves in PH or PL, in the present work, we implement a Laplacian filtration procedure on our weighted Laplacian matrix to construct a family of subgraphs based on an accumulation threshold. The function of the weighted Laplacian matrix is the following:


(4.10) $$\begin{array}{lcr} & \begin{array}{r} L=(l_{ij}),l_{ij}=\left\{ \begin{array}{cc} l_{ij}, & i\neq j,i,j=1,\ldots ,n\\ l_{ii}=-\sum_{j=1}^{n}l_{ij}. & \end{array}\right. \end{array} & \end{array}$$

For i≠j, let $l_{\text{max}}=\text{max}(l_{ij})$, $l_{\text{min}}=\text{min}(l_{ij})$ and $d=l_{\text{max}}-l_{\text{min}}$. We set the kth PL $L^{k},k=1,\ldots ,p$:


(4.11) $$\begin{array}{r} L^{k}=(l_{ij}^{k}),l_{ij}^{k}=\left\{ \begin{array}{cc} 0, & \text{if}\;l_{ij}\leq (k/p)d+l_{\text{min}}\\ -1 & \text{otherwise}\\ l_{ii}^{k}=-\sum_{j=1}^{n}l_{ij}^{k}. & \end{array}\right. \end{array}$$

Additionally, figure 4d describes the topological representation of point clouds through Vietoris–Rips complexes with different filtration radius α. The behaviours of Betti-0 and Betti-1 of PH over filtration are illustrated as the red lines in figure 4e. In contrast, the lowest non-zero eigenvalues ($\lambda_{0}^{\alpha ,0}$ and $\lambda_{1}^{\alpha ,0}$) of PL over the same filtration are illustrated by blue lines in figure 4e. PL captures not only all topological changes but also homotopic geometric evolution during the filtration. The topological representation of point clouds in regular octagon can be found in electronic supplementary material, figure S8.

## Data Availability

The datasets and codes used in the paper are shared on Zenodo [55]. Supplementary material is available online [56].
